# Basic Surveillance Parameters Improve the Prediction of Delayed Cerebral Infarction After Aneurysmal Subarachnoid Hemorrhage

**DOI:** 10.3389/fneur.2022.774720

**Published:** 2022-03-02

**Authors:** István Csók, Jürgen Grauvogel, Christian Scheiwe, Jürgen Bardutzky, Thomas Wehrum, Jürgen Beck, Peter C. Reinacher, Roland Roelz

**Affiliations:** ^1^Department of Neurosurgery, Medical Center – University of Freiburg, Faculty of Medicine, University of Freiburg, Freiburg, Germany; ^2^Department of Neurology, Medical Center – University of Freiburg, Faculty of Medicine, University of Freiburg, Freiburg, Germany; ^3^Department of Stereotactic and Functional Neurosurgery, Medical Center – University of Freiburg, Faculty of Medicine, University of Freiburg, Freiburg, Germany; ^4^Fraunhofer Institute for Laser Technology, Aachen, Germany

**Keywords:** subarachnoid hemorrhage, cerebral vasospasm (CVS), delayed cerebral infarction, prediction, risk chart

## Abstract

**Background:**

To establish a practical risk chart for prediction of delayed cerebral infarction (DCI) after aneurysmal subarachnoid hemorrhage (aSAH) by using information that is available until day 5 after ictus.

**Methods:**

We assessed all consecutive patients with aSAH admitted to our service between September 2008 and September 2015 (*n* = 417). The data set was randomly split into thirds. Two-thirds were used for model development and one-third was used for validation. Characteristics that were present between the bleeding event and day 5 (i.e., prior to >95% of DCI diagnoses) were assessed to predict DCI by using logistic regression models. A simple risk chart was established and validated.

**Results:**

The amount of cisternal and ventricular blood on admission CT (**Hijdra sum score**), early **sonographic vasospasm** (i.e., mean flow velocity of either intracranial artery >160 cm/s until day 5), and a simplified binary **level of consciousness** score until day 5 were the strongest predictors of DCI. A model combining these predictors delivered a high predictive accuracy [the area under the receiver operating characteristic (AUC) curve of 0.82, Nagelkerke's *R*^2^ 0.34 in the development cohort]. Validation of the model demonstrated a high discriminative capacity with the AUC of 0.82, Nagelkerke's *R*^2^ 0.30 in the validation cohort.

**Conclusion:**

Adding level of consciousness and sonographic vasospasm between admission and postbleed day 5 to the initial blood amount allows for simple and precise prediction of DCI. The suggested risk chart may prove useful for selection of appropriate candidates for interventions to prevent DCI.

## Introduction

Delayed cerebral infarction (DCI) affects ~20–30% of patients with aneurysmal subarachnoid hemorrhage (aSAH) and contributes considerably to the high morbidity and mortality of this condition (1). The amount of extravasated blood during aneurysm rupture is a central risk factor for DCI (2). Furthermore, poor clinical condition on admission, age, race, smoking, hypertension, and many other factors may be linked to the risk of patients for DCI (3). Yet, at the individual level, DCI prediction remains difficult. This interferes with optimal allocation of preventive, diagnostic, and therapeutic measures to counteract DCI.

Delayed cerebral infarction risk prediction models that are currently available commonly rely on parameters that are available on hospital admission (4–6). However, existing scores attain only moderate predictive accuracy.

Since DCI does not occur during the first days after an aSAH, observation characteristics during the pre-DCI period may be used to increase predictive accuracy (7, 8). Transcranial Doppler (TCD) ultrasonography represents the most commonly applied routine diagnostic for cerebral vasospasm (CVS) in neurocritical care (9). Pathological increase in arterial flow velocity in TCD is associated with an increased risk for DCI (10) and timing of TCD changes seem to be crucial (11–14). In addition, the level of consciousness potentially represents an important lead to DCI risk stratification (3).

Therefore, we set out to establish a simple-to-use risk prediction model based on classical admission characteristics as well as TCD and level of consciousness surveillance during the first days after an aSAH.

## Methods

Study data are available upon reasonable request and in accordance with European data protection rules.

### Study Population

This retrospective study took place in the neurosurgical department of a tertiary referral center. This study was approved by the independent ethics committee of our medical center (reference number: 575/16) and informed consent was waived.

The study adheres to the Strengthening the Reporting of Observational Studies in Epidemiology (STROBE) guidelines for reporting of observational studies (15).

We included all consecutive patients with an aSAH, confirmed by CT or MRI, who were admitted to our neurosurgical service over a 7-year period (September 2008 to September 2015). Patients with admission delay (admission ≥4 days after the aSAH) and early mortality (≤4 days after the aSAH) were excluded. This period was chosen because electronic documentation of daily TCD and level of consciousness were available from September 2008. The period ends when methods of intracranial blood clearance were implemented in our department (16).

Intensive care was provided in accordance with current guidelines and without changes throughout the treatment period (17). In particular, preventive and therapeutic interventions for DCI were not modified.

### Data Collection

#### Baseline Data

Age, sex, the Charlson Comorbidity Index (18), history of hypertension, clinical condition on admission [World Federation of Neurosurgical Societies (WFNS) grade], location and size of the ruptured aneurysm, aneurysm treatment method, and intracerebral hemorrhage were recorded as baseline data.

#### Delayed Cerebral Infarction

The primary endpoint was DCI, which refers exclusively to DCI visualized by cranial imaging. We did not apply a compound endpoint (commonly termed as delayed cerebral ischemia), consisting of both the delayed infarction and delayed neurological deterioration. Available imaging studies were assessed for DCI according to the imaging criteria suggested by Vergouwen et al. (19). Imaging studies and clinical data were reviewed by an interdisciplinary board of aSAH specialists consisting of a neurologist (JBa), a neurosurgeon (CS), and a neuroradiologist. Board rating adjudicated cerebral infarcts to DCI or early cerebral infarcts (e.g., due to aneurysm rupture or medical procedures or other causes). New cerebral infarction on CT or MRI within 6 weeks after aSAH or on the latest CT or MRI before death within 6 weeks, not present on the CT or MRI scan between 24 and 48 h after early aneurysm occlusion and not attributable to other causes such as surgical clipping or endovascular treatment, was classed as DCI. Hypodensities on CT resulting from hydrocephalus, ventricular catheters, or intraparenchymal hematomas were not regarded as DCI (19). The time between aSAH onset and an imaging diagnosis of DCI as defined above was recorded (DCI latency).

#### Blood Amount

The amount of subarachnoid blood on the admission CT scan was classified by using the semiquantitative Hijdra sum score, ranging from 0 to 30 for blood amount in the basal cisterns and from 0 to 12 for the blood amount within the four brain ventricles (20). The modified Fisher scale was also recorded (21).

#### Transcranial Doppler Ultrasonography

Daily TCD assessment and documentation were performed by the treating physicians at the time of aSAH therapy. Documentation of the maximum mean flow velocity (MFV) of either intracranial artery was retrieved. According to our clinical practice and cutoffs proposed in the pertinent literature, sonographic CVS (sCVS) was defined as MFV of ≥160 cm/s of either intracranial artery (22).

#### Level of Consciousness

Daily routine clinical documentation of the level of consciousness was reviewed by two physicians (IC + RR). To obtain a simple, clinically oriented, and dichotomous grading of the level of consciousness, we followed the proposal of Kupas et al. (23) and used the following binary transformation of the Glasgow Coma Scale (GCS): judgment was made for each day whether a patient “follows commands” or “does not follow commands”. In patients with aphasia, “follows commands” was rated, if the patient was at least able to make contact with the treating physician. Patients with deep sedation were rated “does not follow commands”. To maintain simplicity and clinical applicability, we avoided consciousness scores of higher complexity (e.g., GCS) that feature poor interrater reliability (24) and are particularly difficult to apply in intubated patients (25, 26).

#### Development and Validation Cohort

Patients were randomly (using the random numbers function, Microsoft Excel) allocated to the three groups: groups 1 and 2 were merged and used for development of a DCI prediction model. Group 3 was used for the model validation. For calibration of the model (i.e., accordance of predicted and observed DCI rates), the validation cohort was randomly split into three blocks. For each block, predicted and observed DCI rates were statistically compared.

### Statistical Analysis

Baseline characteristics are presented as means ± SD, medians with interquartile range (IQR), or frequencies (%), as appropriate. Differences in baseline characteristics between patients from the development and validation cohorts were assessed by using the Mann–Whitney *U* test, the Fisher's exact test, or the Pearson's chi-squared test, as appropriate.

Delayed cerebral infarction was the primary endpoint of all the statistical analyses.

By using the development cohort only, the univariate regression analyses of available variables were calculated to identify potential risk factors for DCI.

Variables reaching a significance level of *p* < 0.2 were then included in the multivariate logistic regression model. Backward stepwise variable elimination was performed to identify independent predictors of DCI.

The discriminative capacity of the predictive model was described by the area under the receiver operating characteristic (AUC) curve. The coefficients of the multivariate regression analysis were used to calculate predicted DCI risks for every point increase in the Hijdra sum score and either value of sCVS and “follows commands” to establish the DCI risk chart. Jamovi version 1.2.27 (www.jamovi.org) statistical software was applied for statistical analyses.

## Results

### Baseline Data

Table 1 summarizes the baseline data of the development and validation cohorts. Two hundred and eighty-three patients were included in the development cohort and 134 patients were included in the validation cohort. DCI occurred in 55 patients (19.4%) of the development cohort and DCI occurred in 29 patients (21.6%) of the validation cohort. Both the cohorts were similar for all the clinical characteristics.

**Table 1:** Patient, aSAH, and treatment characteristics in a consecutive aSAH population randomly distributed into the prediction development (two-thirds) and validation (one-third) cohorts.

	**Development cohort**	**Validation cohort**	***p*-value**
Number of patients	283	134	
Delayed cerebral infaction (DCI), *n* (%)	55 (19.4)	29 (21.6)	0.60
Latency of DCI, days after ictus, mean (SD)	12.4 (8.6)	13.1 (6.1)	0.23
DCI prior day 5 after aSAH, *n* (% of DCI cases)	3 (5)	0 (0)	0.55
**Patient characteristics**			
Female, *n* (%)	193 (68)	87 (65)	0.51
Age at diagnosis, years, mean (IQR)	55.2 (46–64)	56.6 (48–64)	0.28
Charlson Comorbidity Index, median (IQR)	1 (0–2)	0 (0–2)	0.21
Arterial hypertension, *n* (%)	105 (37)	51 (38)	0.85
**aSAH characteristics**			
Admission WFNS-Grade, *n* (%)			0.37
1	85 (30)	39 (29)	
2	51 (18)	20 (15)	
3	14 (5)	7 (5)	
4	37 (13)	11 (8)	
5	96 (34)	57 (43)	
**Modified fisher scale**, ***n*** **(%)**			
0	3 (1)	3 (2)	0.89
1	29 (10)	12 (9)	
2	22 (8)	11 (8)	
3	67 (24)	33 (25)	
4	161 (57)	75 (56)	
**Hijdra score, median (IQR)**			
Total	16 (9–24)	15 (8–23)	0.75
Ventricles	2 (0–4)	2 (0–4)	0.96
Cisterns	13 (6–20)	12 (5–21)	0.84
Intracerebral hemorrhage, *n* (%)	76 (27)	43 (32)	0.30
**Location of ruptured aneurysms**, ***n*** **(%)**			
ICA	53 (19)	19 (14)	0.48
MCA	61 (22)	36 (27)	
ACA	123 (43)	60 (45)	
PCA	46 (16)	19 (14)	
Aneurysm size (mm) median (IQR)	6.0 (4.0–8.4)	6.8 (4.5–9.2)	0.15
**Aneurysm treatment**, ***n*** **(%)**			
Clip	139 (49)	61 (46)	0.49
Coil	144 (51)	73 (54)	

*ACA, anterior cerebral artery; aSAH, aneurysmal subarachnoid hemorrhage; DCI, delayed cerebral infarction; ICA, internal carotid artery; IQR, interquartile range; MCA, middle cerebral artery; PCA, posterior circulation artery; WFNS, World Federation of Neurosurgical Societies*.

### Variable Derivation

**Figure 1:**
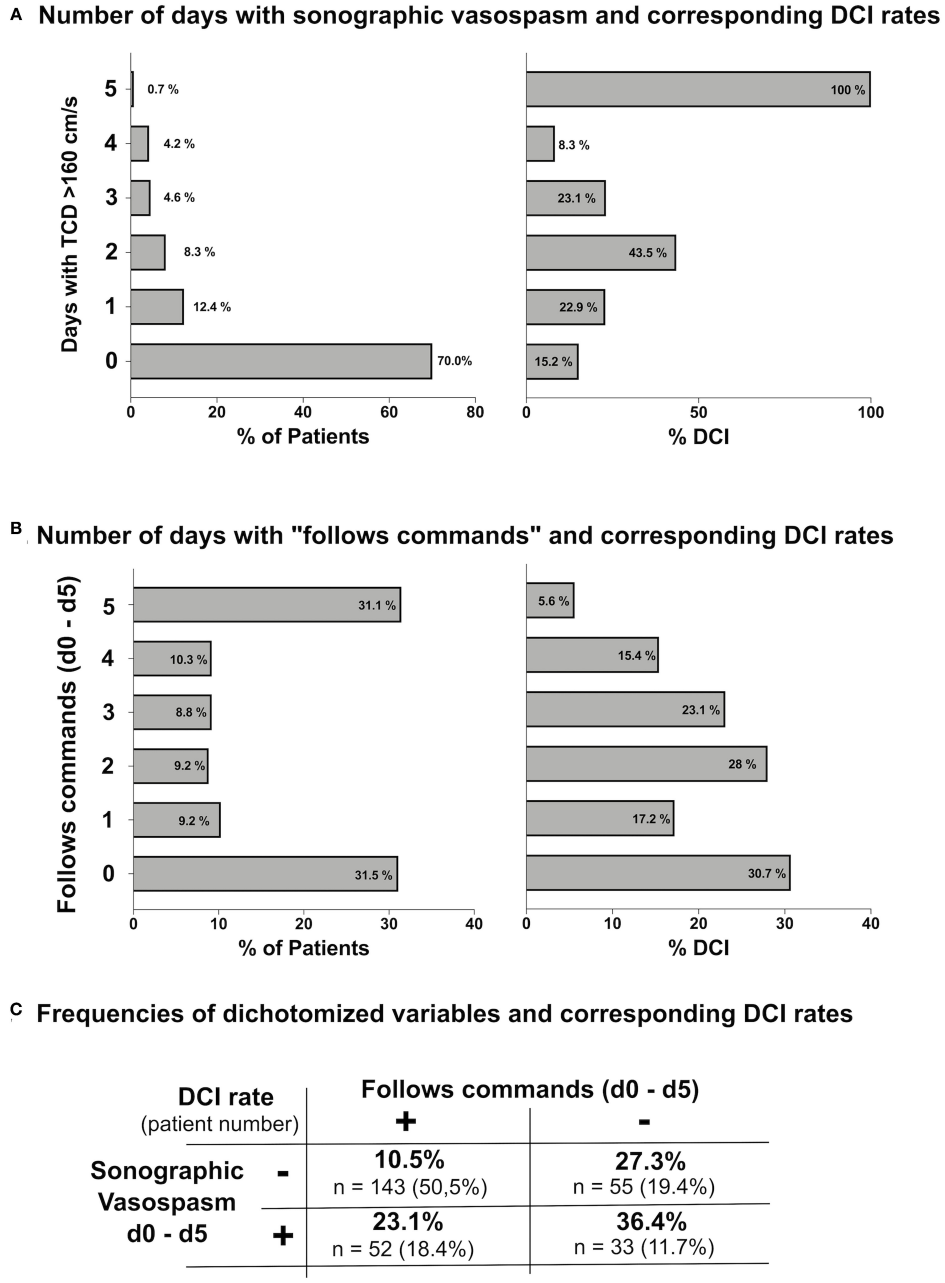
**(A)** Number of days with sonographic cerebral vasospasm (sCVS) until postbleed day 5 and corresponding delayed cerebral infarction (DCI) rates. **(B)** Number of days with “follows commands” until postbleed day 5 and corresponding DCI rates. **(C)** Summary of DCI rates observed when sCVS and “follows commands” were applied as dichotomous variables.

The development cohort was interrogated for associations between: (a) the **number of days with sCVS** and the DCI rate and (b) the **number of days with “follows commands”** and the DCI rate (Figures 1A,B). Thirty percent of patients featured sCVS on at least one of the first 5 days after aSAH. Patients with sCVS had a higher rate of DCI compared to patients without sCVS (28.2 vs. 15.2%, *p* = 0.013). However, no correlation between the number of days with sCVS and an increasing DCI rate was observed. Thirty-one percent of patients “followed commands” on all the 5 days after aSAH and a low DCI rate of 5.6% occurred in these patients. The DCI rate was higher in patients who did not “follow commands” on at least one of the first 5 days postbleed (30.7 vs. 13.9%, *p* < 0.001). Variable rates of DCI were observed with an increasing number of days with “follows commands”.

Given the lack of a correlation between the observed number of days with both the sCVS and “follows commands” and the DCI rate, both the variables were dichotomized for further analyses: “any number of days with sCVS” vs. “no sCVS” and “any number of days with follows commands” vs. “no day with follows commands." Figure 1C summarizes observed the DCI rates corresponding to these dichotomized variables.

### Uni- and Multivariable Predictors of DCI: Development of a Predictive Model

**Figure 2:**
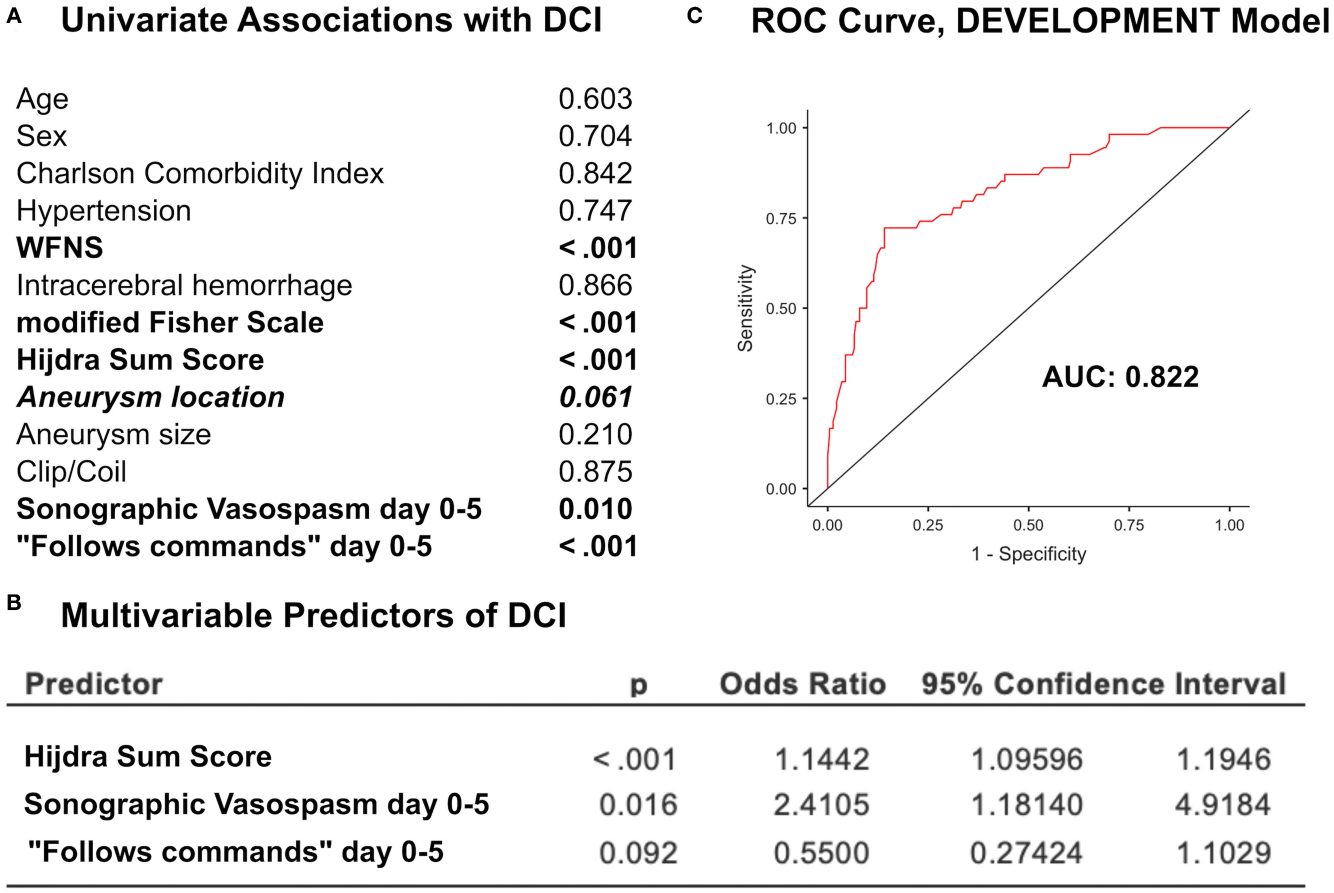
**(A)** The univariate analysis of predictors of DCI in the development cohort. **(B)** The multivariate logistic regression analysis of independent predictors of DCI. **(C)** The receiver operating characteristic (ROC) curve of the predictive model including the Hijdra sum score, sCVS, and “follows commands” showed the area under the curve (AUC) of 0.822.

The univariate logistic regression analysis showed statistically significant associations between DCI and the following variables: WFNS grade, modified Fisher scale, the Hijdra sum score, sCVS, and “follows commands” (Figure 2A). The multivariate logistic regression analysis identified the Hijdra sum score and sCVS as independent predictors of DCI (*p* < 0.05). “Follows commands” featured a near-significant association [odds ratio (OR) 0.55, 95% CI 0.27–1.10, *p* = 0.092]. “Follows commands” was kept in the model, since it substantially improved the model fit (Nagelkerke's *R*^2^ from 0.32 to 0.34) (Figure 2B). Predictive accuracy of the model was the AUC of 0.82 (Figure 2C).

### Model Validation

**Figure 3:**
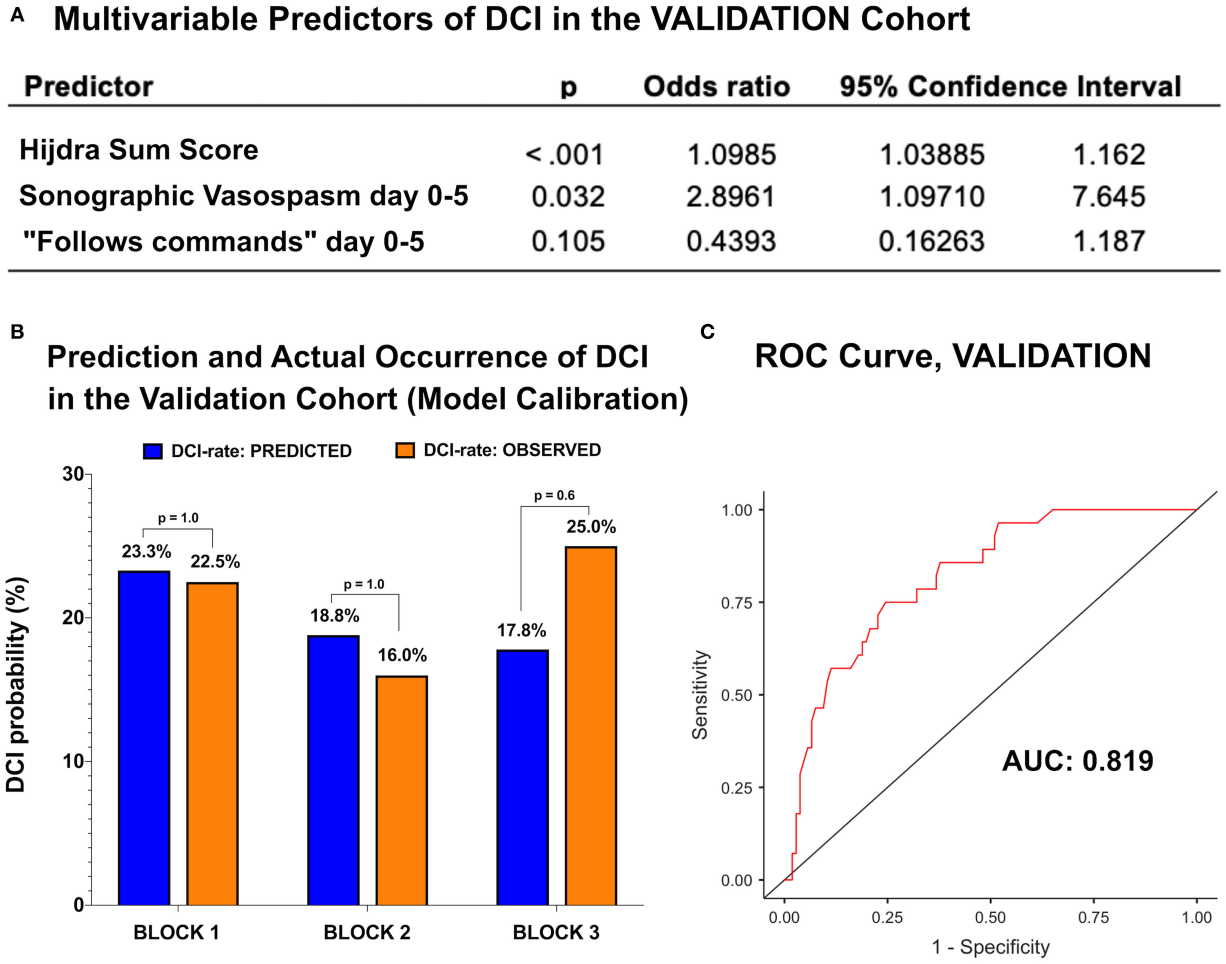
**(A)** The multivariate logistic regression analysis of independent predictors of DCI in the validation cohort. **(B)** The validation cohort was split in three blocks. Predicted and observed DCI rates were congruent in all the three blocks. **(C)** The ROC curve of the development model showed the AUC of 0.819.

The model was applied to the validation cohort and an excellent accordance with the development data was observed. The OR and 95% CIs of the Hijdra sum score, sCVS, and “follows commands” were highly congruent (Figure 3A). The validation cohort was randomly split into three blocks. In each block, statistical agreement of predicted and observed DCI rates was maintained (Figure 3B). The discriminatory performance for DCI was high (AUC 0.82) and the model fit (Nagelkerke's *R*^2^: 0.30) was good (Figure 3C).

### Delayed Cerebral Infarction Risk Chart

**Figure 4:**
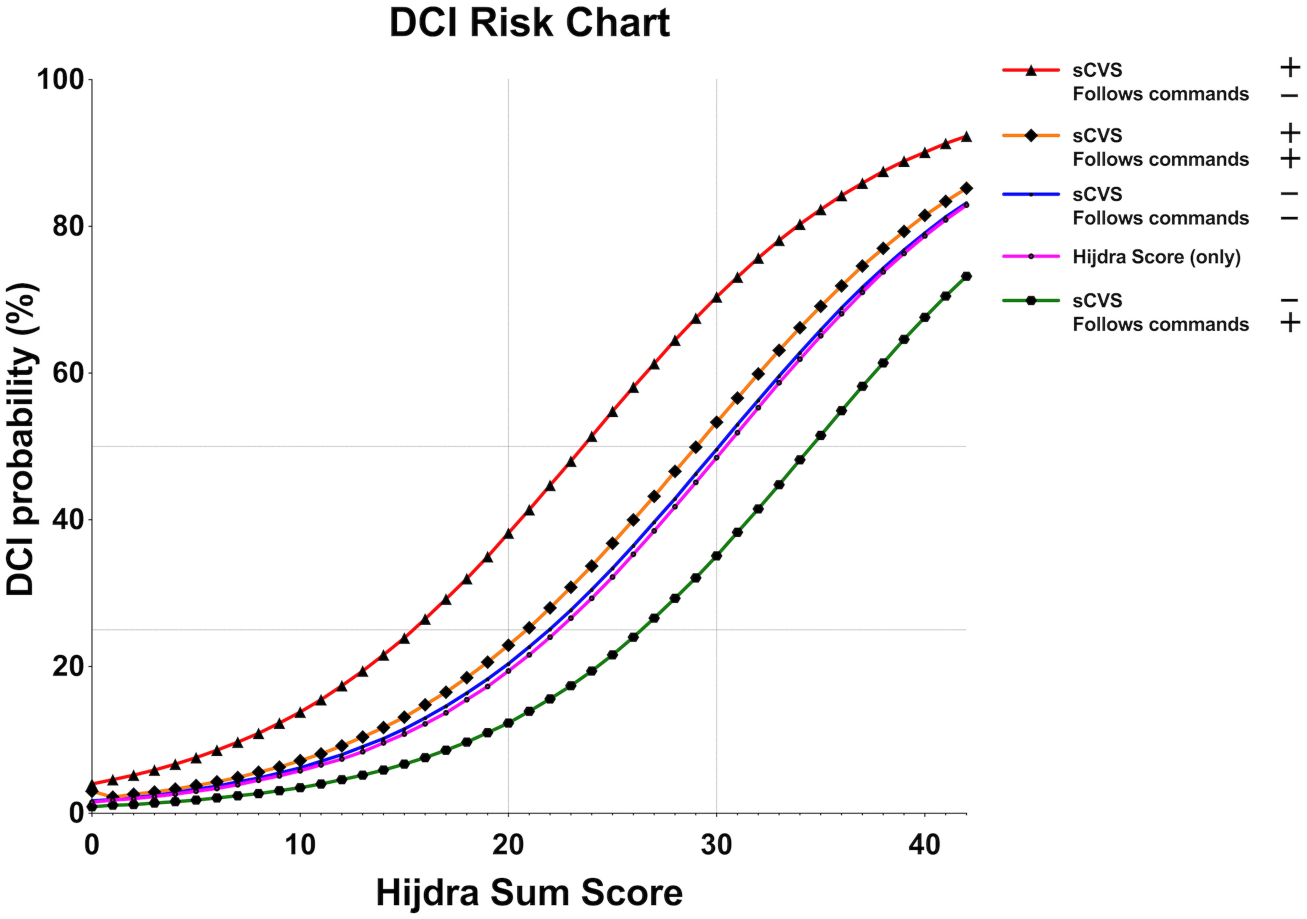
Predicted DCI risk relative to the baseline Hijdra sum score. sCVS and the level of consciousness—simplified to a binary parameter: “follows commands/does not follow commands” —represent important modifiers to the individual DCI risk profile. A sharp increase in DCI risk applies to patients who feature sCVS and “does not follow commands” on the first 5 days after aneurysmal subarachnoid hemorrhage (aSAH) (red line). In contrast, the baseline DCI risk according to the blood amount (the Hijdra sum score, pink line) is considerably reduced in patients who follows commands and lack sCVS (green line). If one modifier is favorable and the other is unfavorable, the DCI risk is neutralized to the baseline risk according to the Hijdra sum score.

A DCI risk chart was created on the basis of the development model. The x-axis of the chart is represented by the Hijdra sum score. For every point increase in the Hijdra sum score, DCI risks were calculated for all the four possible categories of the binary variables sCVS and “follows commands.” We included the “Hijdra sum score only” predictive model (pink line) to demonstrate the impact of both the observation variables for DCI risk estimates (Figure 4). A strong upward shift of the DCI risk curve resulted, if both the observation variables were unfavorable (occurrence of sCVS on either day 0–5 and lack of “follows commands” on every day 0–5) (red line). Conversely, the DCI risk dropped drastically below the baseline “Hijdra sum score only” risk, if both the variables were favorable (green line). Opposing values for sCVS and “follows commands” neutralized the impact of each other and led to DCI risks near the “Hijdra sum score only” baseline (yellow and blue lines).

## Discussion

We created a simple-to-use risk chart to accurately predict DCI in patients with aSAH. The only information needed is the baseline amount of extravasated blood (recorded by the semiquantitative Hijdra sum score) and daily information on sCVS and a simplified level of consciousness assessment (“follows commands: yes/no”) until postbleed day 5. Validation of the risk chart confirmed a high predictive accuracy (AUC 0.82).

Currently, available DCI prediction approaches mainly rely on baseline data and achieve only moderate predictive accuracy (4–6, 8). DeRooij et al. suggested a score based on cisternal and ventricular blood amount, the WFNS grade, and age that yielded the AUC of 0.63 (4). The predictive accuracy of a score based on the WFNS and the modified Fisher grade equally yielded the AUC of 0.63 (5). We hypothesized that taking surveillance parameters into account until the onset of the critical phase of DCI (prior to postbleed day 6) could improve DCI prognostication and, thereby, assist clinical decisions to guide aSAH therapy. Our focus was to improve DCI prognostication by using *simple means*. We believe that incorporating complex or technically demanding parameters into prognostic models considerably limits their clinical utility. Accordingly, only parameters that are collected in clinical routine were eligible for the creation of the model.

The amount of blood on the initial cranial CT is an undisputed risk factor for DCI (27). The Hijdra sum score is a simple and quick semiquantitative classification of the blood amount that features high interrater reliability (28). It has repeatedly been found to deliver a relatively accurate DCI prognostication that outweighs categorical blood amount classifications (e.g., Fisher scale/modified Fisher scale) (29). These findings are confirmed in this study.

We chose sCVS as an observation variable, since TCD represents the only non-invasive method for vasospasm surveillance recommended by current guidelines (30). Despite all the technical limitations (poor sensitivity, low interrater reliability, and inadequate insonation in 10% of patients), occurrence of sCVS is clearly associated with DCI. (10, 31) This study confirms the prognostic relevance of sCVS for DCI prognostication. We observed that a binary grading “sCVS on either of the first 5 days postbleed: yes/no” represented a strong modifier of the baseline DCI risk. Considering the number of days with sCVS did not further improve DCI prognostication. We believe that specific training of physicians and technicians could further increase the value of vasospasm surveillance by TCD.

Poor clinical status has been linked to an increased DCI risk in numerous previous investigations. Commonly, the level of consciousness on admission (typically in the form of the WFNS or Hunt and Hess grade) is considered (4–6). Our analysis shows that the observation of the level of consciousness during the first days after an aSAH outweighs the admission status for DCI prognostication. We argue that the true severity of the bleeding event is better represented by the level of consciousness throughout the first days compared to hospital admission. Epilepsy, hydrocephalus, sedation, and many other factors may skew the initial judgment of the neurological condition of patients with aSAH. To comply with clinical routine and maintain simplicity of DCI prognostication, we avoided the use of complex neurological grading scales. The GCS features a relatively poor interrater reliability and is of limited use in intubated patients (25, 26). Accordingly, we adopted a simplified and validated version of the GCS that dichotomizes the neurological status into “follows commands: yes/no” (23).

The derived DCI risk chart is extremely simple to apply in all the patients with an aSAH. It is intended to improve the identification of patients at risk for DCI and, accordingly, assist clinical decisions to implement DCI diagnostics and treatments.

### Study Limitations

This study is subjected to the general constraints of retrospective analyses. In particular, measurement bias for DCI due to lack of standardization of imaging across patients cannot be ruled out. Further retrospective analyses may always be subjected to researcher bias. We tried to exclude any potential bias on the study endpoints by independent and external assessments. The primary endpoint, DCI, was assessed by an independent and blinded rating board consisting of experienced physicians who were not involved in the treatment of patients.

## Conclusion

Initial blood amount and surveillance of sonographic vasospasm and “follows commands/does not follow commands” until postbleed day 5 allow accurate DCI prediction in patients with aSAH. On this basis, we created a simple risk chart to improve DCI prognostication.

## Data Availability Statement

The raw data supporting the conclusions of this article will be made available by the authors upon reasonable request and in accordance with European data protection rules.

## Ethics Statement

The studies involving human participants were reviewed and approved by Ethics Committee, Medical Center – University of Freiburg, Germany. Written informed consent for participation was not required for this study in accordance with the national legislation and the institutional requirements.

## Author Contributions

IC and RR contributed to the study conceptualization, data collection, analysis and interpretation, statistical analyses, visualization, and drafting of the manuscript. JG contributed to the data curation, data collection and interpretation, and reviewed the manuscript for important intellectual content. CS contributed to the data curation, study supervision, interpretation of data, and reviewed the manuscript for important intellectual content. JBa and TW contributed to the interpretation of data and reviewed the manuscript for important intellectual content. JBe contributed to the study conceptualization, data curation, project administration, and reviewed the manuscript for important intellectual content. PR contributed to the study conceptualization, collection and interpretation of data, and drafting of the manuscript. All authors contributed to the article and approved the submitted version.

## Funding

RR was funded by the Berta-Ottenstein-Programme for Advanced Clinician Scientists, Faculty of Medicine, University of Freiburg, Germany. The article processing charge was funded by the Baden-Wuerttemberg Ministry of Science, Research and Art and the University of Freiburg in the funding programme Open Access Publishing.

## Conflict of Interest

PR received personal fees and non-financial support from Boston Scientific (Marlborough, USA), personal fees and travel support and honoraria for lectures from Brainlab (Munich, Germany), and a research grant from the Fraunhofer Society (Munich, Germany). The remaining authors declare that the research was conducted in the absence of any commercial or financial relationships that could be construed as a potential conflict of interest.

## Publisher's Note

All claims expressed in this article are solely those of the authors and do not necessarily represent those of their affiliated organizations, or those of the publisher, the editors and the reviewers. Any product that may be evaluated in this article, or claim that may be made by its manufacturer, is not guaranteed or endorsed by the publisher.
